# Entropy-Induced Self-Assembly of Colloidal Crystals with High Reflectivity and Narrow Reflection Bandwidth

**DOI:** 10.3390/e21020180

**Published:** 2019-02-14

**Authors:** Xiaoyi Chen, Hongbo Xu, Mengyao Pan, Jiupeng Zhao, Yao Li, Ying Song

**Affiliations:** 1 School of Chemistry and Chemical Engineering, Harbin Institute of Technology, Harbin 150001, China; 2 Center for Composite Materials and Structure, Harbin Institute of Technology, Harbin 150001, China

**Keywords:** soft matter, colloidal crystals, self-assembly, entropy-induced

## Abstract

Cracks and defects, which could result in lower reflectivity and larger full width at half maximum (FWHM), are the major obstacles for obtaining highly ordered structures of colloidal crystals (CCs). The high-quality CCs with high reflectivity (more than 90%) and 9.2 nm narrow FWHM have been successfully fabricated using a fixed proportion of a soft matter system composed of silica particles (SPs), polyethylene glycol diacrylate (PEGDA), and ethanol. The influences of refractivity difference, volume fractions, and particle dimension on FWHM were illuminated. Firstly, we clarified the influences of the planar interface and the bending interface on the self-assembly. The CCs had been successfully fabricated on the planar interface and presented unfavorable results on the bending interface. Secondly, a hard sphere system consisting of SPs, PEGDA, and ethanol was established, and the entropy-driven phase transition mechanism of a polydisperse system was expounded. The FWHM and reflectivity of CCs showed an increasing trend with increasing temperature. Consequently, high-quality CCs were obtained by adjusting temperatures (ordered structure formed at 90 °C and solidified at 0 °C) based on the surface phase rule of the system. We acquired a profound understanding of the principle and process of self-assembly, which is significant for preparation and application of CCs such as optical filters.

## 1. Introduction

Yablonovitch and John have proposed a kind of material with a periodic dielectric structure—photonic crystals (PCs) [1,2]—whose enormous influence is comparable to that of semiconductor technology. Research on PCs has been published twice by *Science* magazine as the world’s major advance in science [3,4]. Self-assembly technique of colloidal crystals (CCs) is considered to be the most promising approach for the preparation of large-area PCs [5]. Aryana et al. pointed out that self-assembly of colloidal clusters with anisotropic building blocks have extended the library of available nanoscale ordered multicompound structures [6]. CCs are three-dimensional ordered periodic structures fabricated by minor particles dispersed uniformly in a solution [7]. Traditionally, the action of gravity [8,9], centrifugal force [10], pressure [11], surface tension [12,13], electric force [14], or magnetic force [15] create conditions (concentration, temperature, fluidity, uniformity, and stability over time), under which the forces of attraction prevail over the repulsive forces on average. These attractive forces could make the directional movement of mono-dispersed particles, leading to the formation of a two-dimensional or three-dimensional ordered structure of colloidal crystal (CC) materials. In science, matter is divided into basic soft and hard materials [16]. Thermal motion and entropy dominate the motion and transformation of soft matter materials due to the weak interactions of the structural units (in the range of kT).

The suspension of mono-dispersed particles is considered as representative of a soft matter system [17]. A fundamental characteristic of soft matter is the large non-linear response to external forces. When CCs are attained under action of external forces, the transformation of the system is non-linear so that internal stress among the particles is produced and released by the appearance of cracks or dislocations. Cracks and defects are the major obstacles for obtaining perfectly ordered structures of CCs. So far, various assembly methods and material systems have been used to avoid cracks and to boost the assembly quality. Garbin et al. summarized strategies to accomplish enhanced regulation over interfacial self-assembly of nanoparticles based on a literature review [18]. Although the assembly quality has been improved by adjusting synthetic parameters such as evaporation rate, pressure, and temperature; the cracks are still inevitable in an evaporation process based assembly [19,20,21,22]. The cracks and defects make the reflectivity of CCs decrease and the reflection bandwidth increase, which is not conducive to getting higher sensitivity and signal-to-noise ratio of optical devices. The soft matter with a particular mesoscopic structure possesses self-assembly characteristics, which is an essential rule of nature creation. Because the precursors of butterfly wings [23], peacock feathers [24], and hummingbird feathers [25] in nature are all soft matter, the formation of their periodic structures rely on the self-assembly behavior under the action of entropy. Recently, Casey et al. developed binary CCs composed of polymer microspheres, which generate characteristic, reversible attractions between two kinds of microspheres, pulled together through DNA bridges [26]. Zanjani et al. discussed computationally the self-assembly of tetrahedral and octahedral clusters, and cubic mediated through “bond spheres” which dock with the clusters at characteristically dominant sites [27]. Wang et al. presented spontaneously growing double-diamond (or B32) crystals which include an appropriate diamond structure by a simple method, using DNA to conduct the self-assembly procedure [28]. Ducrot et al. demonstrated that spheres and tetrahedral clusters, interacting by a DNA-mediated short-range attractive interaction, form superlattice of pyrochlore sublattices and interpenetrating diamond by self-assembly [29]. Zanjani et al. emphasized some superstructures assembled by binary systems, which are composed of “merged” or “sintered” single spheres and tetrahedral clusters [30]. Ducrot et al. designed a new principle which allows the formation of otherwise unattainable structures, furthermore this principle based on programmed nearest-neighbor DNA-mediated interactions and preassembled components of the desired superstructure [31]. Aryana et al. proposed new multicompound superstructure phases that could self-assemble from binary mixtures of building blocks, and the connectivity landscape of diverse shapes of colloidal molecules was investigated [32]. Poly-(methyl meth-acrylate) (PMMA) particles sterically stabilized with poly-(methyl methacrylate)-graft-polyhydroxystearic acid (PMMA-g-PHSA) have been employed as a hard sphere model to phase transitions and crystallization based soft matter [33,34,35,36], which is favorable for avoiding cracks and defects in the process of forming the CCs. As a result, it is beneficial to obtain CCs with high reflectivity. However, it is almost impossible to acquire CCs with narrower full width at half maximum (FWHM) that is less than 10 nm.

Herein, we used the fixed proportional silica particles (SPs), polyethylene glycol diacrylate (PEGDA), and ethanol to establish a soft matter system and discussed its self-assembly behavior induced by entropy. Firstly, we creatively designed a closed system (on the planar interface) in which CCs had been successfully fabricated using this soft matter system. Secondly, we proposed a hard sphere system (consisting of SPs, PEGDA, and ethanol) and confirmed that the emergence of ordered CC structures depended on the phase transition induced by entropy. Owing to the completely spontaneous phase-transition process of the system, we could gain CCs with diverse reflectivity and different full FWHM of the reflection spectrum by controlling temperatures in the crystallization process. Consequently, the high-quality CCs with higher reflectivity and narrower FWHM of the reflectance spectrum were acquired. This research would significantly promote the application of PCs in the field of optical devices such as optical filters.

## 2. Materials and Methods

### 2.1. Preparation and Characterization of SPs

PEGDA (Mn = 250), 2-hydroxy-2-methylpropiophenone (96%), and the standard sample of ethanol (99.9%) were purchased from Sigma-Aldrich. Tetraethylorthosilicate (TEOS, 98%) and aqueous ammonium (NH_3_•H_2_O, 28%) were purchased from Aladdin. The ethanol (99.7%) was purchased from Sinopharm Chemical Reagent Co. Ltd. (Shanghai, China).

SPs were synthesized using a modified Stöber method. The particle dimensions of the SPs were characterized by SEM, the crystalline state was determined by XRD, and the surface group was examined by the Brook infrared spectrometer. Polydispersity index (PDI) of SPs was determined through the dynamic light scattering method. 

### 2.2. The Self-Assembly of Soft Matter on the Planar Interface and the Bending Interface

After being dried to constant weight at 60 °C, 40 µL of SP powders were dispersed in 2.0 mL anhydrous ethanol using a centrifuge tube (capacity 5 mL). Constant weight means that the weight difference (weighing twice) is less than 0.2 mg (considering the random errors). The mixture was subsequently treated uniformly by ultrasonic waves. After adding 60 µL of PEGDA into the mixture and dispersing uniformly by vortex mixer, the mixture was heated to a constant weight at 90 °C and formed a fixed proportional soft matter system. Then, after being cooled down to 25 °C, 30 µL of the mixture was taken out and added into another 10 mL round bottom centrifuge tube (the bending interface), being set for 20 min after sealing. Then 0.6 µL of 2-hydroxy-2-methylpropiophenone was permeated into the mixture, and then they were solidified for 1 min with ultraviolet (UV) light. At the same temperature, another 30 µL of the mixture was taken out and placed on a square hydrophobic glass container (poly methyl methacrylate) with a size of 15 × 7.5 × 0.3 mm (the planar interface). A cover glass was used to keep the system closed, being set for 20 min and forming metastable silica particle CCs in PEGDA. Then 0.6 µL of 2-hydroxy-2-methylpropiophenone was permeated into the mixture, which was solidified for 1 min with UV light.

### 2.3. Effect of Temperature on the Self-Assembly Behavior of the Soft Matter System

The influences of different temperatures on the reflectivity and FWHM were discussed. The treatments such as mixing, heating, etc. were described in the previous paragraph. After achieving the constant weight at 90 °C, one of four centrifuge tubes containing a certain amount of split charging mixture was placed in an ice-water mixture (at 0 °C) and the other three in constant-temperature water baths separately (at 30, 60, and 90 °C). The above four samples (taking out 30 µL of each mixture) were separately placed on four square hydrophobic glass containers (polymethyl methacrylate) with a size of 15 × 7.5 × 0.3 mm (the planar interface), then four cover glasses were separately used to keep each system closed, which were pre-treated to the corresponding temperatures (0, 30, 60, and 90 °C), being set for 20 min and forming metastable silica particle CCs in PEGDA. Then 0.6 µL of 2-hydroxy-2-methylpropiophenone was permeated into each of the above samples, which were solidified for 1 min with UV light. Relevant characterizations of the samples were performed.

### 2.4. The Preparation of CCs with High Reflectivity and Narrow Reflection Bandwidth

The treatments such as mixing, heating, etc. were described in the previous paragraph. After achieving the constant weight of the mixture at 90 °C, 30 µL of the mixture was placed on a square hydrophobic glass container (polymethyl methacrylate) with a size of 15 × 7.5 × 0.3 mm (the planar interface), and then a cover glass was used to keep the system closed, which was preprocessed to 90 °C, being set for 20 min and forming metastable silica particle CCs in PEGDA. The mixture was cooled slowly to 25 °C, cooled down further, and maintained at 0 °C. Then 0.6 µL of 2-hydroxy-2-methylpropiophenone was permeated into the mixture, which was solidified for 1 min with UV light. UV light solidifying conditions: the wavelength was 365 nm and the power was 4.8 mW/cm^2^.

### 2.5. Determination of Optical Characteristics of CCs

The optical photographs were taken using a Canon 70D. The reflection spectra were measured using an Ocean Optics Maya 2000 Pro spectrometer coupled to a six-around-one reflection/back scattering probe, where both the incident and reflective angles were fixed at 0°.

### 2.6. Determination of Ethanol

Ethanol was measured by an external standard method of gas chromatography using a 7820A gas chromatograph (Agilent, America) and a hydrogen flame ionization detector.

## 3. Results and Discussion

### 3.1. Characterization of Synthetic SPs

**Figure 1:**
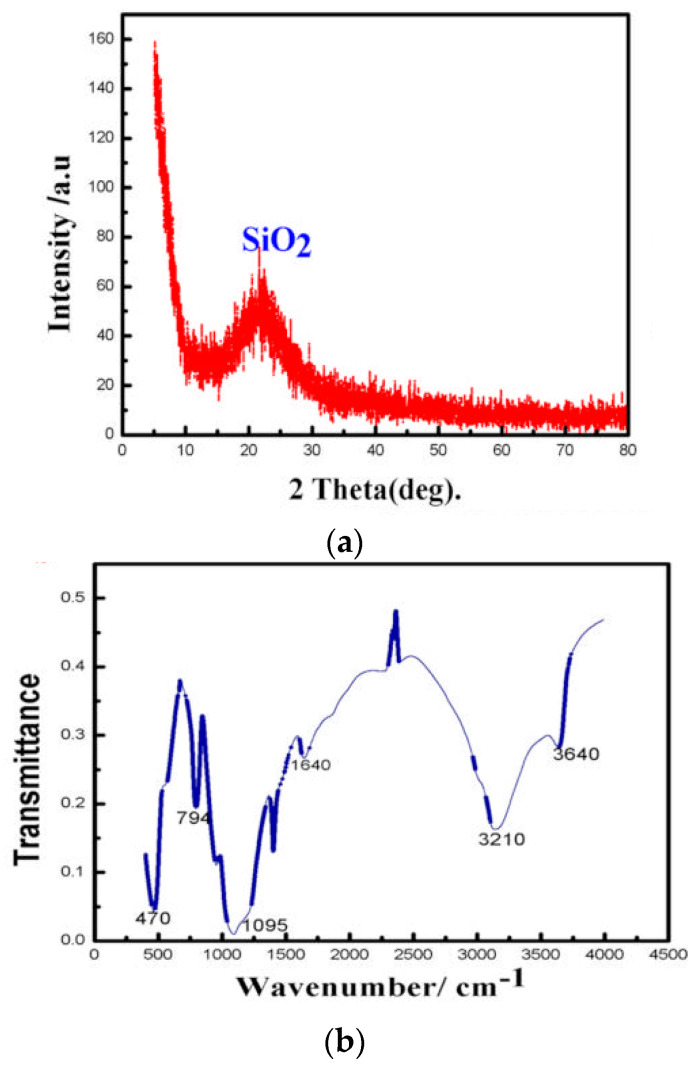

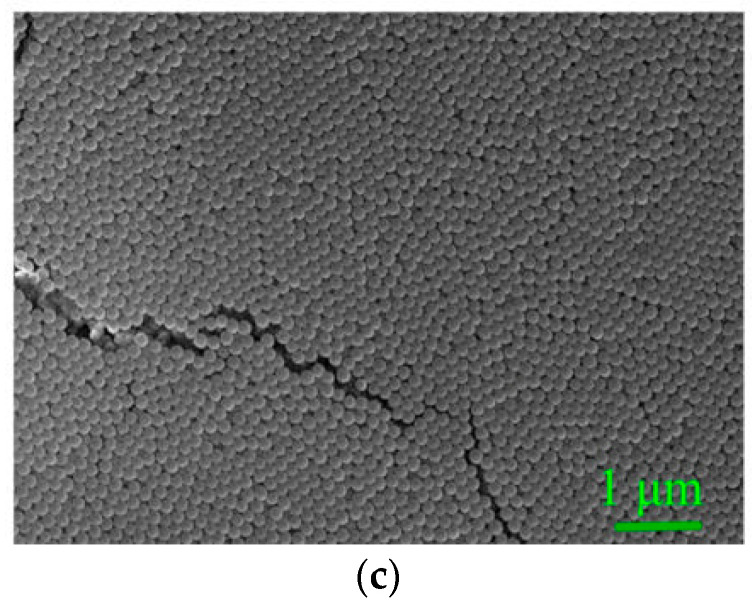
Basic characteristics of the synthetic silica particles (SPs). (**a**) XRD pattern of SPs. (**b**) IR spectrum of SPs. (**c**) SEM image of SPs.

The X-ray diffraction (XRD) pattern and the scanning electron microscopy (SEM) image of SPs are illustrated in Figure 1a,c, respectively. The main component is identified as silica by comparing Figure 1a with the standard XRD pattern, and the as-prepared SPs are amorphous. The dimensions (average diameter is 186 nm) are uniform (PDI is 0.013 from Figure S1); the spherical morphology and dispersivity of SPs are also favorable. The infrared (IR) spectrum, as shown in Figure 1b, of the SPs indicates that the absorption peak of free hydroxyl appears at 3640 cm^−1^ and the associating hydroxyl at 3210 cm^−1^; the strong and wide absorption bands at 1095 cm^−1^ correspond to the anti-symmetric-stretching peaks of the Si–O–Si bond; the peaks at 470 and 794 cm^−1^ are the symmetric-stretching peaks of the Si–O–Si bond.

### 3.2. Determination of Composition and Proportion of the Three-Component Soft Matter

**Figure 2:**
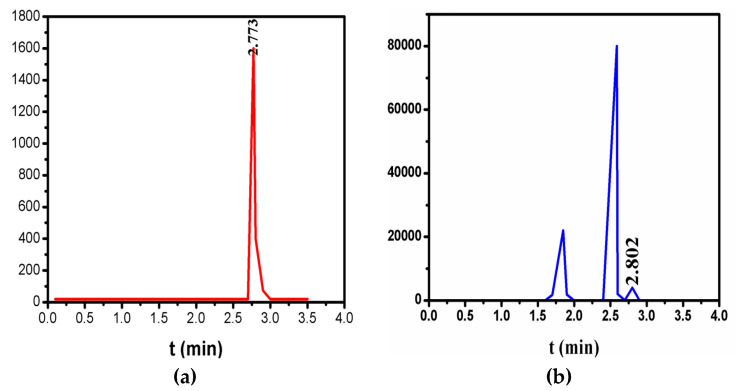
(**a**) Gas chromatogram (GC) of the ethanol standard sample. (**b**) GC of the three components after evaporating to constant weight at 90 °C and removing SPs from this system.

SPs (40 µL) were added into 2 mL of anhydrous ethanol, which were mixed with 60 µL of PEGDA (Mn = 250). We evaluated the ultimate components of the system to demonstrate the presence and function of ethanol after evaporating the system to constant weight at 90 °C. The retention time of gas chromatogram (GC) of the ethanol standard sample is 2.773 min, as shown in Figure 2a. Figure 2b shows the GC of the three-component system after evaporating to constant weight at 90 °C and removing SPs from this system. It is obvious that the retention time (2.80 min) corresponds to the characteristic peak of ethanol by comparing with the standard sample, as shown in Figure 2a. The content of residual ethanol is 11.95 µL by quantitative calculation of the peak area. When the system reached constant weight, for the liquid two-component system composed of ethanol (11.95 µL) and PEGDA (60 µL), a homogeneous single phase was formed because of the mutual dissolution between ethanol and PEGDA. Therefore, in a closed system, the ratio and total volume of ethanol and PEGDA remained constant according to the phase rule. In addition, the content of SPs was determined in this study, and the proportion of the three components remained constant in the subsequent closed system experiments. SPs include hydrophilic silicon hydroxyl and PEGDA contains hydrophobic groups. Ethanol acted as a dispersing aid that the hydroxyl groups interacted with the silicon hydroxyl of SPs and the alkyls with the hydrophobic groups of PEGDA. Photographs of the dispersed system are shown in Figure S2 and Figure S3. SPs were steadily dispersed in the PEGDA system under surface-activated action of ethanol, as shown in Figure S2.

### 3.3. Self-Assembly of the Soft Matter System on the Planar Interface

**Figure 3:**
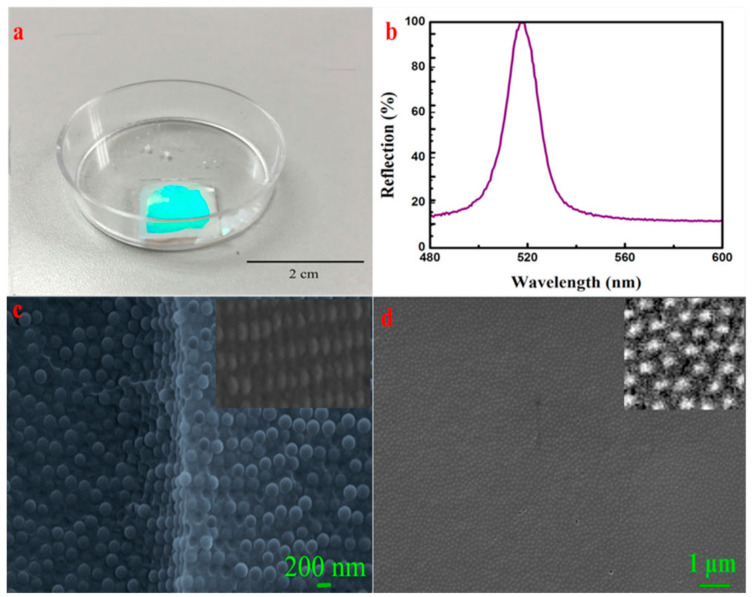
Ordered colloidal crystal (CC) structure appeared on the planar interface using the fixed proportion of a soft matter system. (**a**) Optical photograph of the CCs, (**b**) reflection spectrum of the CCs, (**c**) SEM image of the cross-section of the CCs, (**d**) surface SEM image of the CCs.

The system automatically crystallizes on the planar interface. The optical photograph presents green iridescent colors, as shown in Figure 3a. The reflection spectrum is observed from Figure 3b, and the reflection center wavelength is 515 nm which approaches the theoretical calculation value (510 nm) in the Supplementary Materials. According to the results of the theoretical calculation, as shown in Figure S4 to Figure S9, the FWHM of obtained CCs is less than 2 nm. However, there may exist various inevitable slight differences in the lattice orientations within the acquired CCs, therefore, the actual FWHM (10.3 nm) is larger than that of the theoretical calculation. The SEM images of the cross-section and the surface are depicted in Figure 3c,d, respectively. All these results illustrate that the particles occupying each lattice point formed the ordered CCs and constructed a face-centered-cubic (FCC) structure (reflection center wavelength of theoretical calculation is 510 nm in Supplementary Materials, which is reasonably in agreement with experimental data, whereas reflection center wavelength of the theoretical calculation of the hexagonal close-packed structure is 442 nm). Consequently, the reflection peak and iridescent colors could be revealed. Figure 3d shows the surface SEM image of CCs with scarcely any cracks, and as a result, the self-assembly of soft matter is favorable for obtaining high-quality CCs.

### 3.4. Formation Mechanism of the Ordered CC Structure Induced by Entropy on the Planar Interface

**Figure 4:**
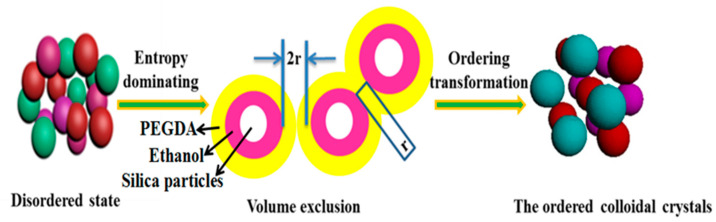
Schematic diagram of the forming process of ordered a face-centered-cubic (FCC) structure using the soft matter system on the planar interface. PEGDA: polyethylene glycol diacrylate.

As a soft matter system, the self-assembly of the three-component system occurred at a planar interface. The self-assembly process was a kind of nucleation and growth according to the phase separation kinetics. The mechanism of formation process of the ordered CCs is shown in Figure 4. As shown in Figure S10, from which we can see that the SPs in the initial state of the three-component system are disordered. The final state of the three-component system is shown in Figure 3c,d, which indicates that the SPs are arranged in an FCC structure.

Figure 4 illustrates the spontaneous formation process of an ordered state of the system. For suspension (the fixed proportion of a soft matter system composed of SPs, PEGDA, and ethanol), it is possible to model hard sphere behavior when the solid particles are confined to spherical objects of sizes in the colloidal range [37]. The hard sphere system composed of SPs, PEGDA, and ethanol assembled three-dimensional CCs through an excluded-volume effect. In this procedure, the phase transition was spontaneous and induced by entropy increase; the internal energy of the system barely contributed to the phase transition. The reduction of free energy of the system was derived from the increase of entropy, which is the driving force of colloidal ordering [38,39,40,41,42]. Entropy acted as a drive action that rejected SP aggregation and accomplished solidification phase change from the fluid to the solid. Thus, common defects of CCs (such as cracks induced by action of gravity, centrifugal force, and pressure etc.) were effectively averted, which made it possible to acquire high-quality CCs.

A monodisperse hard sphere system, as a simple condensed state structure, has been extensively discussed [43,44,45,46]. As the volume fraction of SPs increases, the system experienced a first-order phase transition driven purely by entropy and the calculation results demonstrate that the solid crystal structure may be an FCC structure or a hexagonal close-packed (HCP) structure. In addition, the free energy between each particle of the FCC structure is lower than the free energy between each particle of the HCP structure (the free energy difference is about 10^−3^ J) [47,48,49], therefore, the FCC structure is the most stable state of thermodynamics. The FCC structure is more stable than the HCP structure (the highest volume fractions of SPs in these two structures are both 74%).

In the actual colloid system, particles inevitably possess certain polydispersity that is defined as the ratio of the variance to average value of particle sizes. PDI of particle sizes could significantly affect the thermodynamics and dynamics behaviors of hard sphere systems [43,50,51,52,53]. The difference of free energy between FCC and HCP structures was calculated by the Monte Carlo simulation, which illustrates that the FCC structure is still the most stable under the condition of low PDI [53,54,55]. The CCs obtained in this research are in an FCC structure, which was in full agreement with the theoretical calculation results. In this study, we used the SPs with an average particle size of 186 nm (PDI is 0.013). In a hard sphere system, the solidification phase change from the fluid to the solid could still occur since the low PDI is a minor perturbation to the monodisperse system [43,51,54,55]. When the value of PDI exceeds a certain limit, the single-phase crystals could be diverted into two or more coexisting crystals which still have the same lattice structure with different particle sizes [51,56].

### 3.5. Self-Assembly of the Soft Matter System on the Bending Interface

**Figure 5:**
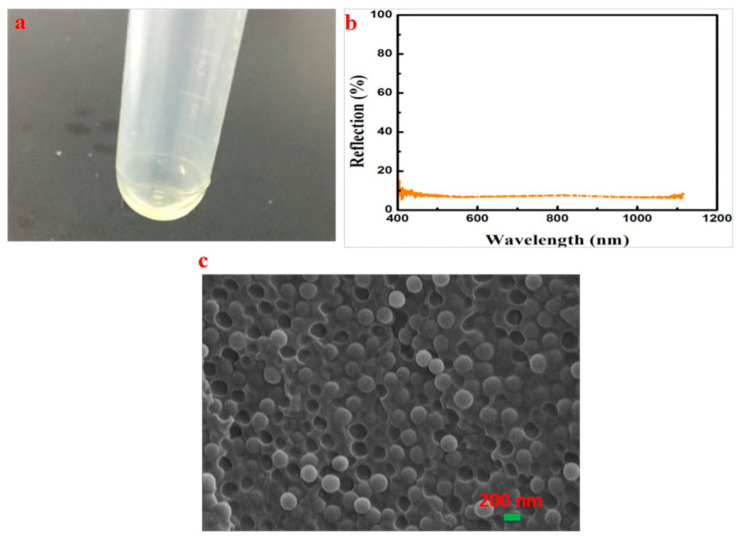
A disordered structure appeared on the bending interface using the fixed proportion of a soft matter system. (**a**) Optical photograph, (**b**) reflection spectrum of the disordered structure, (**c**) SEM image of the cross-section of the disordered structure.

On a bending interface, the CC structure hardly formed in the system composed of SPs, ethanol, and PEGDA. The optical photograph with inconspicuous iridescent colors, as shown in Figure 5a, and the reflection spectrum, as shown in Figure 5b, are displayed. The present state hardly emerged a reflection peak, as shown in Figure 5b, indicating that the soft matter system failed to crystallize. The cross-sectional SEM image, as shown in Figure 5c, demonstrates that the particles in each site of the lattice were disarrayed on the bending interface.

The primary reason is that the self-assembly of the three-component system occurred on the bending interface. According to phase separation kinetics, this is a kind of spinodal decomposition. According to the Laplace equation, additional pressure exists on the bending interface, which induces tremendous structural changes under the action of weak external force, resulting in phase separation of the system and the formation of disorderly bicontinuous structure. Ordered structure may be developed with the increase of radius of curvature on the bending interface, however, even if ordered structure exists, the reflectivity of CCs is less than that on the planar interface. In addition, the FWHM of CCs is larger than that on the planar interface based on the theoretical analysis of the above.

### 3.6. Effect of Temperature on the Self-Assembly Behavior on the Planar Interface

By using the method of plane wave expansion, as shown in Figure S4 to Figure S6, we have predicted the effects of refractive index difference, volume fraction, and particle dimensions of SPs on the FWHM of CCs, as shown in Figure S4 to Figure S9. The theoretical calculation of the maximum FWHM will be less than 2 nm if the refractive index difference is 0.01, the volume fraction of SPs 40%, and the particle dimension below 200 nm. The effects of volume fraction of SPs on the FWHM of the reflection spectrum are illustrated in Figure S8, which shows that the volume fraction of SPs has less influence on FWHM (from 2 nm to 1 nm). Figure S11 indicates that the volume fraction of SPs has a vital influence on the reflection center wavelength. In the two-phase colloid system composed of two kinds of colloidal spheres with the radius R (the larger SPs) and the radius r (the smaller PEGDA spheres), the mixing entropy of the system requires that the larger SPs be evenly distributed in the smaller PEGDA spheres. When the radius difference between the larger SPs and the smaller PEGDA spheres is large enough, the phase separation of the colloidal system will inevitably occur at a certain concentration, i.e., crystallization. The theoretical calculation results of Figure S11 are suitable for the sample preparation because the maximum volume fraction of the SPs in the FCC structure is 74%.

**Figure 6:**
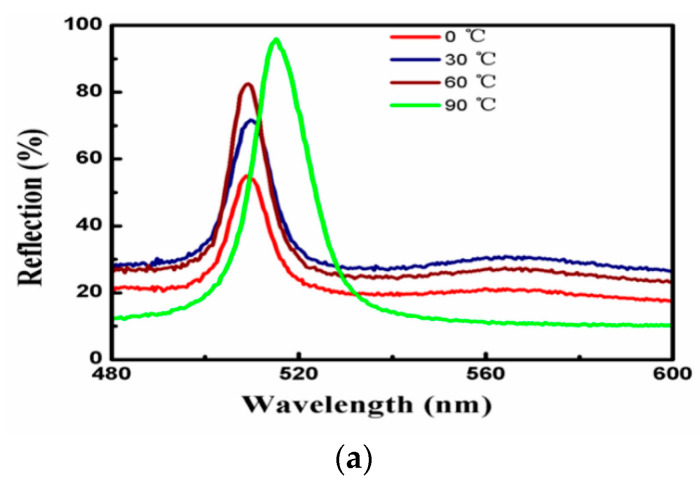

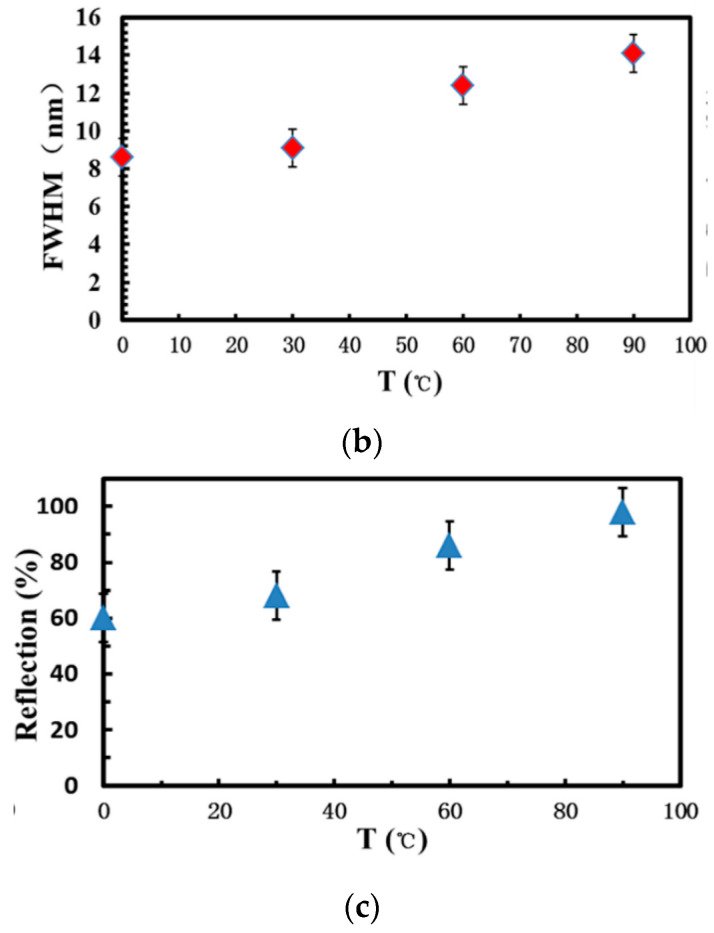
Effects of different temperatures on reflection properties of CCs. (**a**) Reflection spectra of CCs at diverse crystallization temperatures. (**b**) The full width at half maximum (FWHM) of reflectance spectra under various temperatures. (**c**) The reflectivity at different temperatures.

The temperature could affect the quality of the CCs formed by the soft matter, because the fluctuation of temperature could impact the volume exclusion effect of PEGDA and the intensity of Brownian motions of the SPs. CCs with diverse reflectivity and FWHM would be gained by modifying the crystallization temperature. The reflection peaks of CCs at different temperatures are shown in Figure 6a. The influence of temperature on self-assembly of the soft matter system was analyzed as follows.

Emptying effect: as shown in Figure 4, when large particles (SPs) were surrounded by minor particles (PEGDA) with a radius of r, the minor particle (PEGDA) could induce large particles (SPs) to crystallize, making the entropy of minor particles (PEGDA) multiply towards infinity. Osmotic pressure, which can be regarded as the change of free energy per unit volume, is responsible for the emptying effect of macromolecules (PEGDA). The free energy change of the corresponding per unit contact area was calculated according to the relation of the entropy-induced emptying effect, as shown in Equation (1).

(1) $$\Delta F/A=\;c\cdot k_{B}\cdot T\;\times \;2r\;$$

In the above equation, $c\cdot k_{B}\cdot T$ is van ’t Hoff’s law of osmotic pressure, ∆F/A is the reduced value of free energy of per unit contact area, c is SP molecular number density of the suspension, k_B_ is the Boltzmann constant, T is the temperature, and r is the radius of PEGDA.

The analysis results of reflectivity variation in Figure 6b demonstrate that the reflectivity rises with intensifying temperature. The primary causation is considered that with the increase of T, the increasing ∆*F/A* led to the free energy (per unit contact area) decreasing greatly. As a result, the ordered structure area in the unit area increased, which made the reflectivity increase. Nevertheless, Figure 6c depicts that the FWHM of the reflectance spectrum also magnifies with the rise of temperature. The augment of the FWHM of the reflectance spectrum caused by the high temperature may be attributed to the enhancing tendency of the particles deviated from the equilibrium position because of the Brownian motion. Actually, the FWHM of the CCs obtained in this research is more than 8 nm due to slight differences in the lattice orientations. Moreover, the larger the number of CC layers, the increased defects of line and surface, which would also enlarge the FWHM.

The three-component system was a heterogeneous suspension. The proportion of SPs to liquid varies slightly when 30 μL mixture was self-assembled on the planar interface at each time. According to Figure S11, the differences between the ratios of SPs to PEGDA lead to a slight difference in the reflection center wavelength, as shown in Figure 6a.

**Figure 7:**
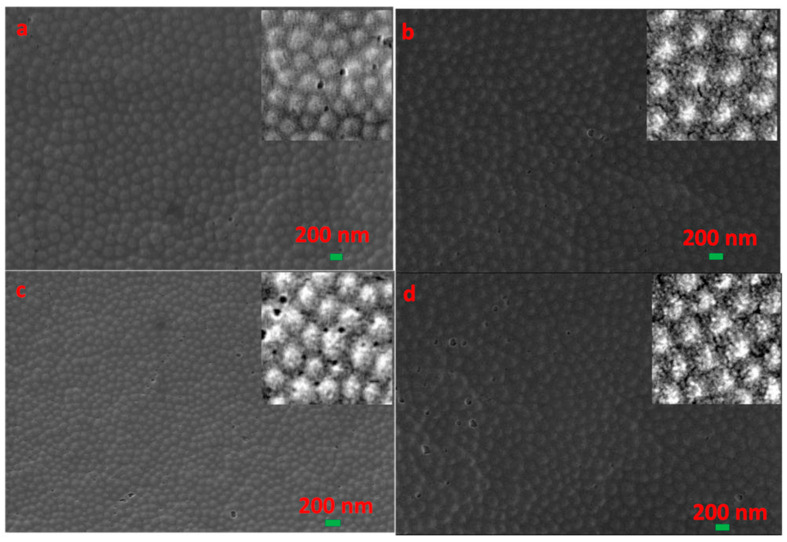
SEM images of CCs formed at different temperatures. (**a**) 0 °C, (**b**) 30 °C, (**c**) 60 °C, (**d**) 90 °C.

It can be seen that the CCs formed at 0, 30, 60, and 90 °C are all FCC structures, as shown in Figure 7. The difference of the ordered area can be clearly seen by fast Fourier transform (FFT) of SEM images, which present highly ordered structure of a larger area with the increase of temperature (consistent with the gradual increase of reflectivity in Figure 6c).

### 3.7. The Preparation of CCs with High Reflectivity and Narrow Reflection Bandwidth

For a polydisperse system, it is assumed that there are C components and P phases in an equilibrium system, and the chemical potential of each component in each phase must be equal to each other on the basis of equilibrium. The degree of freedom of the whole system is by the equation:

(2) F = P(C − 1) + 2 − C(P − 1) = C − P + 2,

which is the phase rule of a polydisperse system [57].

In order to obtain CCs with high reflectivity and narrow FWHM, the surface phase rule of bending interface was analyzed according to the following equation:

(3) f = K − ( g − s ) + 1.

**Figure 8:**
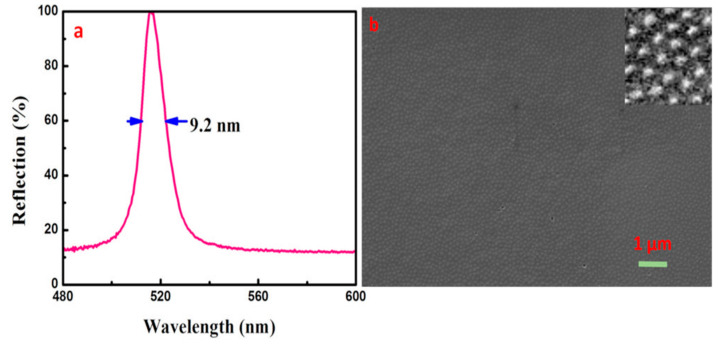
(**a**) Reflection spectrum of the CCs solidified at 0 °C (the ordered structure of CCs was formed at 90 °C). (**b**) SEM image of the CCs solidified at 0 °C (the ordered structure of CCs was formed at 90 °C).

SPs and PEGDA droplets were exposed in the vapor interface, with K = 1, g = s = 1, R = 0, f = 2. There exist two independent degrees of freedom, they may be (T, r_L_) or (T, r_g_) etc. That r_L_ is density of mixed liquids (ethanol and PEGDA), and r_g_ is gas density of mixed liquids (ethanol and PEGDA). Equation (3) is applicable to the bending interface [57]. According to the phase rule, the change of temperature hardly changed the state of the system. That is, in this study, crystallization could not occur on the bending interface during the slow cooling process. The surface phase rule of the planar interface is given by:

(4) f = K − Φ − ( g − s ) + 2,

where K represents the species number, s the interface varieties, Φ the number of phases, g the surface phase number, f the degrees of freedom. Equation (4) is suitable for the planar interface [57], and for insoluble SPs and PEGDA two-component systems (ethanol as dispersing aid), a new phase arose and the formation of CCs with an obvious interface (surface) depended on the phase transformation of the hard sphere system. Here, Φ = 3, g = 4, s = 3, f = 2, the degrees of freedom include temperature (T) and pressure (P). After crystallization, the change of temperature hardly affected the ordered structure of the system according to the phase rule. CCs with different reflectivity and FWHM could be obtained by adjusting temperatures of crystallization and solidifying. The high-reflectivity CCs were obtained at higher temperature, and then the variations of temperature barely converted the ordered state of the CCs. The Brownian motions of SPs were weakened at the lower temperature, which was favorable to the formation of CCs with fewer defects. Figure 8a illustrates the reflection spectrum of CCs which had been solidified at 0 °C (the crystallization of CCs had been accomplished at 90 °C). The SEM image presents FCC colloidal crystals, which are of a higher ordering degree, larger area, fewer defects, and scarcely any cracks, as shown in Figure 8b. Thus, high-quality CCs with the narrower FWHM (reduced from 13.6 nm to 9.2 nm) were obtained, the reflectivity of which was higher than 90%.

## 4. Conclusions

In summary, a hard sphere system was established using a fixed proportional three-component system composed by SPs, PEGDA, and ethanol to produce CCs based on self-assembly behavior of the soft matter. High quality CCs could be fabricated on the planar interface, however, unfavorable results were presented on the bending interface. The influences of refractivity difference, volume fractions, and particle dimension on FWHM were illuminated. The influence of temperature on the self-assembly behavior of CCs was expounded. The FWHM and reflectivity of CCs showed an increasing trend with the increase of temperature. High-quality CCs with high reflectivity and narrow reflection bandwidth were obtained by adjusting temperatures (the ordered structure of CCs was formed at 90 °C and solidified at 0 °C). The enhancement of reflectivity and narrower FWHM of the reflectance spectrum could improve the sensitivity and signal-to-noise ratio of optical devices, which may significantly advance the application of PC devices such as filters.

## Supplementary Materials

The following are available online at http://www.mdpi.com/1099-4300/21/2/180/s1, Figure S1: Size distribution of synthetic 186 nm silica particles (SPs). Figure S2: Optical photograph of stable and uniform dispersion system formed by polyethylene glycol diacrylate (PEGDA) and SPs in ethanol. Figure S3: Optical photograph of inhomogeneous dispersion system formed by PEGDA and SPs without ethanol. Figure S4: Schematic diagram of first Brillouin zone. Figure S5: Schematic diagram of band structure of CCs composed of SPs and PEGDA. Figure S6: Band structure from L to U. Figure S7: Effect of refractivity difference on the FWHM. Figure S8: Effect of volume fraction of SPs on the FWHM. Figure S9: Effect of the particle dimension of SPs on FWHM. Figure S10: SEM image of the disordered SPs in initial state of three-component system when achieving constant weight. Figure S11: Theoretical prediction of the effects of volume fractions of SPs on reflection center wavelengths of CCs.
