# Supplementary material for: Entropy-Induced Self-Assembly of Colloidal Crystals with High Reflectivity and Narrow Reflection Bandwidth
Source: Entropy (Basel). 2019 Feb 14;21(2):180. doi: 10.3390/e21020180 (PMC7514662; doi:10.3390/e21020180)
Supplement: Supplementary file 1 [file entropy-21-00180-s001.zip › supplementary materials.docx]

Entropy-Induced Self-Assembly of Colloidal Crystals with High Reflectivity and Narrow Reflection Bandwidth

Xiaoyi Chen ^1^, Hongbo Xu ^1^, Mengyao Pan ^2^, Jiupeng Zhao ^1,^*, Yao Li ^2,^*, Ying Song ^1^

^1^	School of Chemistry and Chemical Engineering, Harbin Institute of Technology, Harbin 150001, China; cxy84828@126.com (X.C.); iamxhb@hit.edu.cn (H.X.); songy@hit.edu.cn (Y.S.)

^2^	Center for Composite Materials and Structure, Harbin Institute of Technology, Harbin 150001, China; 18297601130@163.com (M.P.)

*	Correspondence: yaoli@hit. edu.cn (Y.L.); jpzhao@hit. edu.cn (J.Z.)

**Figure S1.** Size distribution of synthetic 186 nm silica particles (SPs).

**Comparison of Dispersion States**

**Figure S2.** Optical photograph of stable and uniform dispersion system formed by polyethylene glycol diacrylate (PEGDA) and SPs in ethanol.

**Figure S3.** Optical photograph of inhomogeneous dispersion system formed by PEGDA and SPs without ethanol.

**Prediction of Full Width at Half Maximum (FWHM)**

**Figure S4.** Schematic diagram of first Brillouin zone.

The first Brillouin zone of the face-centered-cubic (FCC) structure is shown in the Figure S4 and the letters Γ, K, and W are for high symmetry points of the structure respectively [1].

The light was resolved by the plane wave expansion method. The photonic band gap was developed by the discontinuous jump of the photon energy in the Brillouin zone boundary. Thus the jump range leaded to the formation of the photonic band gap. Figure S5 demonstrates hybrid band structure of photonic crystals (PCs) (with distinct dielectric constant) composed of SPs and PEGDA materials.

**Figure S5.** Schematic diagram of band structure of CCs composed of SPs and PEGDA.

**Figure S6.** Band structure from L to U.

By using the method of plane wave expansion, we calculated the band structure from the point L (1, 1, 1) to point U (1, 0.25, 0.25) of the Brillouin zone shown in Figure S6. The full width at half maximum (FWHM) of theoretical arithmetic is 2 nm when the refractive index difference is 0.01, SP dimension 200 nm, and the volume fraction of SPs 30%. The relationship between the refractive index difference and the FWHM of theoretical arithmetic is exhibited in Figure S7, which demonstrates a strict linear relationship between the FWHM and the refractive index difference (in the range of 0.003 to 0.03).

**Figure S7.** Effect of refractivity difference on the FWHM.

We calculated the impact of the volume ratio of SPs and PEGDA on the FWHM of reflection spectrum by the plane wave expansion method. SPs with a particle dimension of 200 nm were used to calculate, the setting value of refractivity difference was 0.01, and the volume fraction of SPs was separately 30%, 40%, 50%, 60%, 70%, and 74%. The relationship between the FWHM and the volume fraction of SPs was acquired. Figure S8 shows that under certain conditions, the FWHM decreased from 2 nm to 1 nm with increasing the volume fraction of SPs. The maximum FWHM reached 2 nm when the volume fraction of SPs was 30%.

**Figure S8.** Effect of volume fraction of SPs on the FWHM.

Under conditions of the refractive index difference 0.01 and the volume fraction of SPs 30%, the relationship between the FWHM and the SP dimension was calculated. Figure S9 indicates that the FWHM enlarged from 2 nm to 3.6 nm with increasing the particle dimension. The maximum FWHM reached 3.6 nm when the SP dimension was 300 nm. From the above calculation results, the theoretical calculation of the maximum FWHM will be less than 2 nm if the refractive index difference is 0.01, the volume fraction of SPs 40%, and the particle dimension below 200 nm. Actually, the FWHM of the PCs obtained in this research is more than 8 nm due to various defects among the CCs and slight difference in the lattice orientations.

**Figure S9.** Effect of the particle dimension of SPs on FWHM.

**Figure S10.** SEM image of the disordered SPs in initial state of three-component system when achieving constant weight.

**Prediction of Reflection Center Wavelength**

For the opal PCs, we could approximately obtain the relationship between the reflection center wavelength and the ordered structural parameters [2] using the general Bragg-Snell formula:

$$\text{Κ}\text{λ}\text{ = 2}\text{d}_{\text{h k l}}\text{ }\text{⋅}\left( {\text{n}^{\text{2}}}_{\text{eff }}\text{-}\text{sin}^{\text{2}} \text{β} \right)^{\frac{\text{1}}{\text{2}}}.$$

(1)

Where *λ* is the position of photonic band gap, *K* the diffraction series, *d_hkl_* the interplanar spacing of the ordered structure, *n_eff_* the effective refractive index of PCs, and *β* the incidence angle relative to the normal sample surface.

This research focuses on opal PCs with a face-centered-cubic (FCC) structure. The relationship between interplanar spacing (d_111_) and SP dimension is indicated by:

$$\text{d}_{\text{1 1 1 }}\text{= }\frac{\text{α}}{\sqrt{\text{1}^{\text{2}}\text{+}\text{1}^{\text{2}}\text{+1}^{\text{2}}}}\text{ = }\frac{\text{α}}{\sqrt{\text{3}}}$$

(2)

Where α is lattice constant (here α equals to 1.62). The effective refractive index ($\text{n}_{\text{eff}}$) of the PC structures can be obtained by:

$$\text{d}_{\text{1 1 1 }}\text{= }\frac{\text{α}}{\sqrt{\text{1}^{\text{2}}\text{+}\text{1}^{\text{2}}\text{+1}^{\text{2}}}}\text{ = }\frac{\text{α}}{\sqrt{\text{3}}}$$

(3)

Parameter $\text{n}_{\text{SiO}_{\text{2}}}$ is refractive index (about 1.46) of SPs, *n_fill_* is refractive index of filling material in particle gaps (for PEGDA, *n_fill_* = 1.47), $f_{SiO_{2}}$ is the volume fraction of particles in the PC structure (the volume fraction of SPs was 40%). So the effective refractive index ($\text{n}_{\text{eff}}$) of FCC structure of the PCs is 1.466. Consequently, theoretical calculation result of reflection center wavelength is 510 nm. The density of SPs is 2.6 g/cm^3^ and the density of PEGDA 1.12 g/cm^3^, thus, the density of CCs is 1.71 g/cm^3^.

The volume fractions of SPs were selected as 30%, 40%, 50%, 60%, 70%, and 74%, the difference of the refractive index 0.01, and the particle size 200 nm. The relationship between the different volume fractions of SPs and the reflection center wavelength was obtained by Bragg-Snell formula. Figure S11 indicates that the higher the volume fraction of SPs, the shorter the reflection center wavelength. This is mainly because the lattice constant of FCC structure decreases with increasing volume fraction of SPs. CCs with different reflection center wavelengths could be acquired by adjusting the different volume fraction of SPs.

**Figure S11.** Theoretical prediction of the effects of volume fractions of SPs on reflection center wavelengths of CCs.

References

1. Satpathy, S.; Zhang, Z.; Salehpour, M.R. Theory of photon bands in three-dimensional periodic dielectric structures. *Phys. Rev. Lett*. **1990**, *64*, 1239.
2. Ohtaka, K. Energy bang of photons and low energy photon diffraction. *Phys. Rev. B.* **1979**, *19*, 5057–5067.
